# Circular RNA ZNF277 Sponges miR-378d to Inhibit the Intracellular Survival of *Mycobacterium tuberculosis* by Upregulating Rab10

**DOI:** 10.3390/cells14040262

**Published:** 2025-02-12

**Authors:** Yifan Zhu, Lei Zhang, Zijian Wang, Ting Li, Yingyu Chen, Lu Lu, Han Liu, Delai Kong, Yongchong Peng, Xi Chen, Changmin Hu, Huanchun Chen, Aizhen Guo

**Affiliations:** 1 National Key Laboratory of Agricultural Microbiology, Hubei Hongshan Laboratory, College of Veterinary Medicine, Huazhong Agricultural University, Wuhan 430070, China; zhuyifan@webmail.hzau.edu.cn (Y.Z.); zhanglei2023@mail.hzau.edu.cn (L.Z.); chenxi419@mail.hzau.edu.cn (X.C.);; 2 National Professional Laboratory for Animal Tuberculosis (Wuhan), Ministry of Agriculture and Rural Affairs, International Research Center for Animal Disease, Ministry of Science and Technology, Huazhong Agricultural University, Wuhan 430070, China

**Keywords:** *Mycobacterium tuberculosis*, circ-ZNF277, macrophages, ceRNA, intracellular survival

## Abstract

Circular RNAs (circRNAs) are covalently closed non-coding RNAs formed by back-splicing, lacking a 5′ cap and poly-A tail. They could act as important regulatory factors in the host’s anti-tuberculosis immune process, but only a few have been identified, and their molecular mechanisms remain largely unclear. Here, we identified a novel circRNA, circ-ZNF277, which responds to *Mycobacterium tuberculosis* (*Mtb*) infection in THP-1 cells. Circ-ZNF277 binds microRNA-378d (miR-378d) in vivo. The expression level of circ-ZNF277 affects the clearance of the intracellular *Mtb* in THP-1 cells. Mechanistically, more circ-ZNF277 molecules could absorb more miR-378d, thereby competitively activating the NF-κB signaling pathway, promoting the release of pro-inflammatory cytokines including interleukins IL-1β and IL-6, and tumor necrosis factor-α (TNF-α), and inhibiting the survival of intracellular *Mtb*. Expressing miR-378d or si-Rab10 targeting the transcription of Rab10 could antagonize the effects of overexpression of circ-ZNF277, resulting in the reduced intracellular survival of *Mtb*. In summary, circ-ZNF277 inhibits the intracellular survival of *Mtb* via the miR-378d/Rab10 axis. This finding represents a novel mechanism of circular RNA in regulating host immune responses during *Mtb* infection.

## 1. Introduction

Tuberculosis (TB) is a highly infectious disease caused by *Mycobacterium tuberculosis* (*Mtb*), which remains the leading cause of death from a single infectious agent, accounting for ~1.5 million deaths worldwide annually [1]. During *Mtb* infection, macrophages serve as the primary “battlefield” against *Mtb* [2,3,4,5]. The co-evolution of *Mtb* and macrophages, particularly *Mtb*’s survival within macrophages, influences the progression of TB [6,7]. First, macrophages initiate the immune response against *Mtb* by recognizing and engulfing the pathogens [8]. They encapsulate *Mtb* within a phagosome, which then fuses with a lysosome to form a phagolysosome, releasing antimicrobial substances such as reactive oxygen and nitrogen species via the NF-kB pathway or through sphingomyelinases to eliminate the intracellular *Mtb* [9,10,11,12]. Additionally, for extracellular *Mtb* that evades initial capture, macrophages secrete cytokines and chemokines to recruit and activate immune cells [5,11,13], including NK cells and T cells, forming granulomas to control and clear the infection [14,15,16,17]. However, *Mtb* has evolved various strategies to survive in macrophages [5,18,19]. For example, *Mtb* could modulate hosts immune responses by secreting virulence factors including protein tyrosine phosphatases PtpA [20], eukaryotic-type protein kinase PknG [21], and acidic phosphatase SapM [22], etc. PtpA binds to the host adaptor TAB3 [20,23] to inhibit NF-κB activation and inhibits phagosome-lysosome fusion by dephosphorylating the host protein VPS33B [24,25]. PknG blocks Rab14-GTP hydrolysis by direct interaction, aiding *Mtb* survival in non-acidified autophagosomes within macrophages [21]. SapM interferes with the acidification and maturation of *Mtb*-containing phagosomes and inhibits autophagy induction by interacting with the host mTORC1 complex component [22,26,27]. In recent years, the functional non-coding RNAs, including circular RNAs (circRNAs) and microRNAs (miRNAs), are also found to play critical roles in regulating the interaction between *Mtb* and the host, as well as in the intracellular survival of *Mtb* [28,29,30,31]. However, the molecular mechanisms remain largely unknown.

Circular RNAs form covalently closed circular structures through typical 5′ to 3′ phosphodiester bonds and regulate biological processes by mediating RNA alternative splicing (AS), regulating cis-transcription, acting as competitive endogenous RNAs (ceRNAs), and translating into peptides [32,33]. Accumulating evidence shows that circRNAs regulate the resistance of the host cells during pathogen infection [34,35,36]. During *Mtb* infection or TB development, circRNAs could affect cell polarization, autophagy, and inhibit the intracellular survival of *Mtb* [37,38]. In some cases, circRNAs target microRNAs (miRNAs) and affect their regulatory functions. For example, circRNA-0045474 triggers macrophage autophagy in tuberculosis via the miR-582-5p/TNKS2 axis [39] and circ-TRAPPC6B inhibits the growth of *Mtb* in macrophages and induces autophagy by targeting miR-874-3p [40]. However, the more regulatory roles of circRNAs in *Mtb* infection remain unclear.

MicroRNA-378d (miR-378d) is involved in various biological processes and diseases, including the regulation of cell autophagy and apoptosis, as well as the inhibition of cancer cell growth and invasion, by regulating the expression of genes such as Gli3, SDAD1, and Rab10 [41,42,43,44]. Among these, Rab10 is a member of the small GTPase family, involved in intracellular membrane transport, and has been shown to play a crucial role in cancer progression, Alzheimer’s disease, and tuberculosis [45,46,47,48]. Previously, we found that the expression level of miR-378d affects *Mtb* survival in macrophages. Downregulating the expression of miR-378d could promote the expression of Rab10, aiding in the clearance of *Mtb* in the host [49]. However, whether there are circular RNAs that could regulate the miR-378d/Rab10 pathway remains unclear.

Here, we identified a novel circular RNA, circ-ZNF277, which responds to *Mtb* infection in THP-1 cells. We showed that the increased expression level of circ-ZNF277 enhances the expression of Rab10 by competitively adsorbing miR-378d. This, in turn, activates the NF-κB signaling pathway and promotes the secretion of pro-inflammatory cytokines, such as IL-1β, IL-6, and TNF-α. Thus, this finding represents a novel mechanism of circular RNA in regulating host immune responses during *Mtb* infection.

## 2. Materials and Methods

### 2.1. Bacterial Strains and Cell Culture

The *Mtb* infection experiments were performed as previously described in the animal biosafety level-3 lab (ABSL-3) in Huazhong Agricultural University (Wuhan, China) [49]. The virulent *Mtb* 1458 strain (GenBank accession no. GCA_001855255.1) was cultured in Middlebrook 7H9 broth supplemented with 10% oleic acid-albumin-dextrose-catalase (OADC) (BD PharMingen, San Diego, CA, USA) and 0.05% Tween-80 (Sigma, Burlington, MA, USA) at 37 °C.

The human monocytic cell line, THP-1 (ATCC, #TIB-202), was cultured in RPMI-1640 medium (Hyclone, South Logan, UT, USA) supplemented with 10% FBS (Gibco, Carlsbad, CA, USA). Before transfection and infection, THP-1 cells were incubated in a culture medium containing 40 ng/mL phorbol 12-myristate 13-acetate (PMA) (Sigma) for 12 h to differentiate into macrophages at 37 °C with 5% CO_2_. The Human Embryonic Kidney (HEK) Cells 293T (ATCC, #CRL-3216) were maintained in Dulbecco’s Modified Eagle’s Medium (DMEM) (Hyclone) supplemented with 10% FBS (Gibco).

### 2.2. Cell Infection with Mtb

THP-1 cells were differentiated with PMA, infected with *Mtb* (MOI = 0.1, 1, 5, 10), and incubated for 12 h (defined as −12 h) to enable *Mtb* to sufficiently enter the cells. Then, the cells were washed two times with fresh medium to remove the extracellular *Mtb* [50]. By taking this time point as 0 h, the cells continued to be cultured in the complete medium with 100 μg/mL Gentamicin for destination times (0 h, 12 h, and 24 h). The cells and supernatants were collected by centrifugation for further analysis.

### 2.3. Generate Stable Cell Lines and Cell Transfection

Circ-ZNF277 cDNA was synthesized and cloned into the pCDH-circRNA-GFP vector (Tsingke, Beijing, China). The pCDH-circ-ZNF277 or pCDH-circRNA-NC was then co-transfected with pMD2.G and psPAX2 into 293T cells using JetPRIME (Polyplus, Illkirch, France) according to the manufacturer’s instructions. After 48 h of transfection, the lentivirus was collected and added to well-growing THP-1 cells (1 × 10^6^) along with 10 µg of Polybrene for 24 h. The medium was then replaced with fresh RPMI containing 10% FBS. After 48 h of culture, the fluorescence of the cells was observed; then the cells were centrifuged, collected, and resuspended in fresh 1640 medium containing puromycin (with 10% FBS). The cells were then continuously selected with puromycin-containing medium until cell fluorescence reached 80–90%. The stable circ-ZNF277-expressing THP-1 cell lines was further expanded and used for *Mtb* infection experiments.

The sequences of si-circ-ZNF277, miR-378d mimic, miR-378d inhibitor, siRab10, and their control sequences are shown in Supplementary Table S1 (Tsingke). For siRNA transfection or co-transfection, cells were seeded into 6- or 12-well plates and cultured to a concentration of (2 × 10^6^) or (1 × 10^6^) cells/mL. The cells were then serum-starved and transfected with si-circ-ZNF277, miR-378d mimic, miR-378d inhibitor, siRab10, and their respective control sequences using JetPRIME (Polyplus) according to the manufacturer’s protocol.

### 2.4. Dual Luciferase Reporter Assays

To specifically detect the interaction between circ-ZNF277 and miR-378d, a dual-luciferase reporter assay was performed. Firstly, the TargetScan program (version 7.1) was used to predict the possible interaction region between circ-ZNF277 and miR-378d, and a 200-bp fragment was selected for in vitro synthesis. The circ-ZNF277 wild type (WT) and circ-ZNF277 mutants (Mut1 and Mut2, with at least six discrete nucleotide point mutations at the predicted binding sites) were cloned into the luciferase reporter plasmid psiCHECK-2 (Promega, Madison, WI, USA). According to manufacturer’s instructions, miR-378d mimics or control mimics were co-transfected with circ-ZNF277 WT, circ-ZNF277 Mut1, or circ-ZNF277 Mut2 into 293T cells using JetPRIME (Polyplus, Illkirch, France). Luciferase activity was measured using the Dual Luciferase Reporter Assay System (Promega, WI, USA). Luminescence was measured by a multimode microplate reader (BioTek Synergy 2, Winooski, VT, USA), and the relative luciferase activity was normalized with the ratio of Renilla to Firefly luciferase values.

### 2.5. Ribonuclease R Digestion Assay

The total RNA of 5.0 × 10^6^ PMA-differentiated THP-1 cells was extracted as previously described [49]. Linear RNA ZNF277 was removed and circ-ZNF277 was enriched by Ribonuclease R (RNase R) digestion. Each sample (0.5 to 1 μg) was treated with 20 U RNase R as previously described [51]. The RNase R-enriched RNA was subjected to further experimental analyses.

### 2.6. Reverse Transcription Polymerase Chain Reaction (RT-PCR)

The genomic DNA (gDNA) of THP-1 cells was extracted using the TIANamp Genomic DNA Kit (TIANGEN, Beijing, China). The HiScript III qRT SuperMix (Vazyme, Nanjing, China) was used to synthesize cDNA from 500 ng of the total RNA for circRNA and mRNA expression analysis. Meanwhile, cDNA was synthesized from 1 µg of total RNA using an All-in-one^TM^ miRNA qPCR Detection Kit (GeneCopoeia, Rockville, MD, USA) for miRNA expression analysis. Subsequently, specific divergent primers and convergent primers were designed for the circ-ZNF277, and RT-PCR reactions were performed with cDNA and gDNA. The PCR conditions were as follows: initial denaturation at 95 °C for 30 s; 40 cycles of denaturation at 95 °C for 5 s, annealing at 60 °C for 30 s.

### 2.7. Quantitative Real-Time PCR (qPCR)

The expression levels of circRNA were normalized to the uninfected control, which was set as 1. Similarly, miRNA and mRNA expression levels were normalized to the transfection control, also set as 1. Subsequently, qPCR analysis was performed with an ABI ViiA 7 Real-Time PCR System (ABI, Waltham, MA, USA) and the AceQ qPCR SYBR Green master mix (Vazyme). The relative expression of circRNA and mRNA was normalized using β-actin, while the relative expression of miRNA was normalized using U6. The gene-specific primers (Tsingke) used for RT-PCR and qPCR are listed in Supplementary Table S2, and the data were presented using 2^−ΔΔCt^ [52].

### 2.8. Fluorescence In Situ Hybridization (FISH) and Nucleocytoplasmic Isolation

FISH assays were performed to observe the location of circ-ZNF277 in THP-1 cells. Enhanced Sensitive ISH Detection kit IV (CY3) (Cat no. MK1033, Boster Biological Technology, Wuhan, China) was used for the hybridizations according to the manufacturer’s protocol. Briefly, after prehybridization at 37 °C for 4 h, cell climbing pieces were hybridized with a specific cy3-labeled circ-ZNF277 probe (cy3–5′-TCCTTACTGTCTTTGGAAATCAGC-3′) (Tsingke) at 37 °C overnight and dyed with 4′,6-diamidino-2-phenylindole (DAPI). Slides were photographed with a fluorescence microscope (Olympus FV1000, Hachioji, Tokyo, Japan).

Cytoplasmic and nuclear RNA were separately isolated as previously described [51]. Extracted RNAs were reverse transcribed immediately, and the circ-ZNF277 expression was measured by qPCR analysis.

### 2.9. Pathway Inhibition Assay

A pathway inhibition assay was performed to determine whether the NF-κB or MAPK signaling pathways might modify circ-ZNF277 expression during *Mtb* infection. PMA-differentiated THP-1 cells were treated with inhibitors (MedChemExpress, Monmouth Junction, NJ, USA) of the PI3K (10 µM, HY-143404) ERK (10 µM, SCH772984), JNK (10 µM, SP600125), p38 (10 µM, SB202190), or p65-NF-κB (20 µM, JSH23) related pathways, respectively, for 2 h, and then infected with *Mtb* as previously described [49]. At 12 h PI, the relative expression of circ-ZNF277 was measured using qPCR.

### 2.10. Western-Blot Analysis

The *Mtb*-infected THP-1 cell lysates were separated by 10% or 15% SDS-PAGE gel and transferred to a polyvinylidene difluoride (PVDF) membrane (Millipore Corporation, Bedford, MA, USA). Then, the membranes were blocked with 5% Skim milk in TBST buffer (TBS containing Tween 20) and then incubated at 4 °C overnight with the primary antibodies (shown in Supplementary Table S3) at 1:1000 dilution in TBST solution. The protein band intensities were measured using the Western Bright ECL (Bio-Rad, Hercules, CA, USA), and β-actin was used as an internal reference. Quantitative analysis of bands was performed using Image J software (version 1.51j8, National Institutes of Health, Bethesda, MD, USA).

### 2.11. Enzyme-Linked Immunosorbent Assay (ELISA)

To detect the secretion of pro-inflammatory cytokines (IL-1β, IL-6, and TNF-α) during *Mtb* infection in THP-1 cells with inhibited or overexpressed circ-ZNF277, or in THP-1 cells co-transfected with si-circ-ZNF277 and miR-378d inhibitor, or in THP-1 cells stably expressing circ-ZNF277 and transfected with si-Rab10, the supernatant of *Mtb*-infected THP-1 cells was filtered at designated time points using a 0.22 μm filter. The protein levels of IL-1β, IL-6, and TNF-α were measured according to the manufacturer’s instructions for the ELISA kit (NeoBioscience, Shenzhen, China).

### 2.12. Colony-Forming Units (CFU) Count

After infecting PMA-differentiated THP-1 cells (1 × 10^6^) with *Mtb* (MOI = 10) for 12 h, extracellular bacteria were removed using gentamicin (100 μg/mL). The infected THP-1 cells were then lysed using sterile deionized water for 25 min at specified time points. The lysates were serially diluted and spread onto 7H11 agar plates supplemented with 10% OADC, as previously described [49]. The plates were incubated at 37 °C for three weeks for CFU counting. Each sample was inoculated in triplicate.

### 2.13. Prediction Analysis of circRNAs Targeting miR-378d

To identify circRNAs targeting miR-378d, several prediction algorithm software tools were utilized, including StarBase v2.0 (https://rnasysu.com/encori/ (accessed on 20 December 2019)) and TargetScan 7.1 (http://www.targetscan.org (accessed on 20 December 2019)). Based on our laboratory’s previous circRNA sequencing data, potential circRNAs targeting miR-378d were further identified.

### 2.14. Statistical Analysis

The statistical analysis was performed using GraphPad Prism 9.0 software (La Jolla, CA, USA). The data were presented as the mean ± SEM. Unpaired two-tailed Student *t*-tests or one-way analysis of variance (ANOVA) were performed as appropriate for statistical analyses. The *p* values are indicated in the figures.

## 3. Results

### 3.1. Circ-ZNF277 Exists in the THP-1 Cells and It Interacts with miR-378d In Vivo

**Figure 1:**
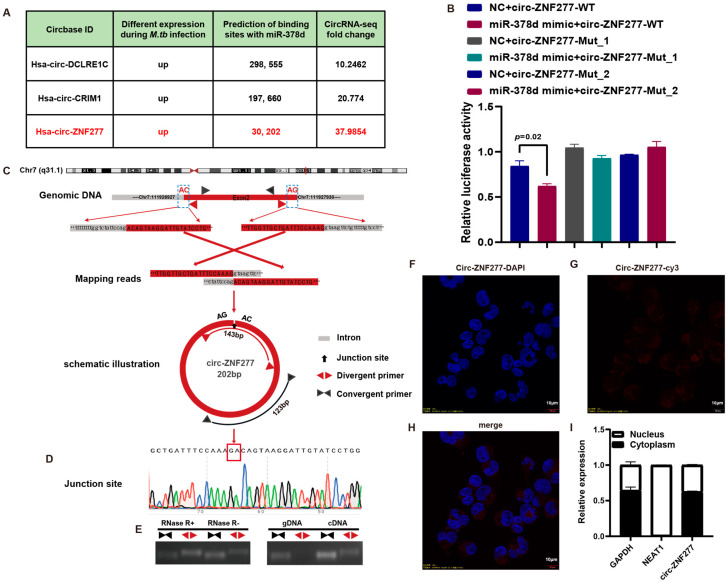
Screening and biological characterization of circ-ZNF277 interaction with miR-378d. (**A**) Screening of circRNAs targeting miR-378d by previous circRNA-seq and two online prediction algorithm software (StarBase and TargetScan); (**B**) Dual-Luciferase Reporter assay was performed in the 293T cells. The wildtype or seed sequence-mutated (mut) versions of circ-ZNF277 were cloned into the psiCHECK-2-Luc vector. The mutations involved at least six base changes (Mut1: TTCTGAAGCACAT to AACTCTAGCTCAA; Mut2: TTCTGAAGCACAT to AAGACTAGGTCAA) at the putative miR-378d-binding sites. psiCHECK-circ-ZNF277 WT, or psiCHECK-circ-ZNF277 Mut1 or psiCHECK-circ-ZNF277 Mut2 were co-transfected with miR-378d mimics or control mimic (NC) into 293T cells, and the lysates were analyzed 24 h later. The ratio represents the Renilla to Firefly (Rluc/Fluc) ratio in miR-378d mimic versus the Rluc/Fluc ratio in the NC mimic; (**C**) Visualization of the circularization mechanism of circ-ZNF277; (**D**) Sanger sequencing identification of the back-splicing junction of circ-ZNF277; (**E**) RNase R digestion assay to detect the RNase R resistance of circ-ZNF277 and ZNF277; Amplification of circ-ZNF277 and ZNF277 in c/gDNA; (**F**–**H**) Detection of circ-ZNF277 distribution in THP-1 cells using a cy3-labeled specific probe through FISH, Scale bar = 10 μm, Magnification, 100×; (**I**) PMA-differentiated THP-1 cells were infected with *Mtb* at an MOI of 10 for 12 h (denoted as 0 h). RNA was extracted from the cells at 12 h post-infection, and the expression of circ-ZNF277 in the cytoplasm and nucleus of THP-1 cells was detected by qPCR, GAPDH (cytoplasm) and NEAT1 (nucleus) serving as internal controls.

Previously, we found that miR-378d, a small non-coding RNA molecule, could affect the survival of *Mtb* in the macrophages. The reduced quantities of miR-378d inhibit the intracellular survival of *Mtb* by increasing Rab10 expression [49]. Further, we wanted to identify the circRNA molecules that could bind miR-378d. In the circRNA-seq data (GEO dataset: GSE248244) from the macrophages infected with virulent and avirulent *Mtb* strains, miR-378d was predicted to interact with the circRNA molecules, including circ-ZNF277, circ-DCLRE1C, and circ-CRIM1 (Figure 1A). Circ-ZNF277 is predicted to be 202-bp in length and it might be generated in the back-splicing process (Figure 1B) for the exon 2 of ZNF277 gene (circBase ID: hsa_circ_0001739) located at chromosome 7q31.1. To test whether circ-ZNF277 is present in the THP-1 cells, the total RNA of the host cells was extracted to generate cDNA library for PCR validation. Using the divergent primer pair (Table S2), a 143-bp DNA fragment was amplified from the cDNA but not from the genomic DNA (gDNA) for detecting circ-ZNF277 (Figure 1E and Supplementary Figure S1). In contrast, a 123-bp DNA fragment was amplified from both the cDNA and gDNA by the convergent primer pair (Figure 1E). This experiment indicated that circ-ZNF277 is indeed present in the THP-1 cells, consistent with the observation that circ-ZNF277 is more resistant to RNAse R than the linear RNA molecules (Figure 1E). In addition, Sanger sequencing confirmed that the 143-bp DNA fragment amplified from the cDNA library contains the junction site of back-splicing (AG|AC) and is identical to the sequence of circ-ZNF277 in the circBase database (Figure 1D).

Further, we detected in vivo interaction between circ-ZNF277 and miR-378d using a dual-luciferase reporter plasmid psiCHECK-2. In the presence of the miR-378d mimics, the luciferase activity in the cells expressing the WT circ-ZNF277 was significantly lower than that from either the cells expressing the mutated circ-ZNF277 (Mut1 or Mut2) or the cells lacking the miR-378d mimics (Figure 1B), suggesting that miR-378d specifically interacts with the WT circ-ZNF277 in vivo.

Next, we examined the cellular localization of circ-ZNF277 in the THP-1 macrophages. The fluorescence in situ hybridization (FISH) experiment was performed to determine the subcellular localization of circ-ZNF277 using a specific cy3 fluorescent probe. The red fluorescence representing the localization of circ-ZNF277 was mainly observed near the nucleus area, suggesting that circ-ZNF277 is localized in the cytoplasm (Figure 1F–H). Additionally, the subcellular fractionation experiment showed that the majority of circ-ZNF277 exists in the cytoplasm (Figure 1I). Collectively, these data indicate that circ-ZNF277 with a circular structure is mainly located in the cytoplasm of THP-1 macrophages.

### 3.2. Circ-ZNF277 Responses to Mtb Infection in THP-1 Cells

**Figure 2:**
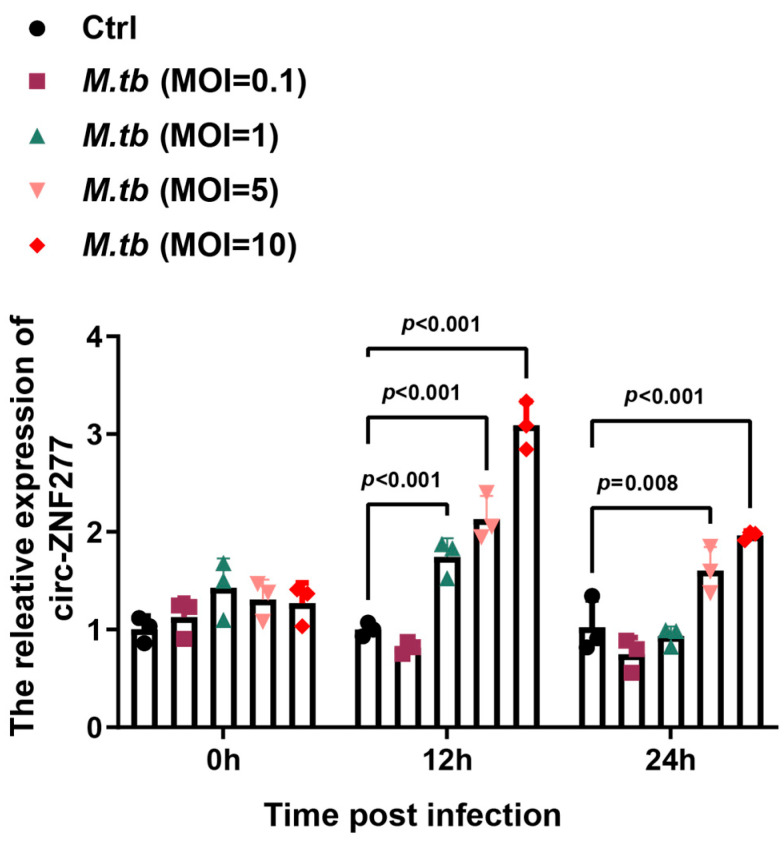
Circ-ZNF277 responses *Mtb* infection in THP-1 Cells. THP-1 cells differentiated with PMA were infected with *Mtb* at different MOIs (0.1, 1, 5, 10) for 12 h. RNA was extracted from the cells at specified time points, and the expression of circ-ZNF277 was detected by qPCR. The data are expressed as mean ± SD. The statistically significant difference was analyzed with ANOVA for more than one comparison.

The circRNA-seq data showed that circ-ZNF277 was significantly upregulated in the THP-1 cells infected with *Mtb* (GEO dataset: GSE248244). To confirm this, we examined the expression level of circ-ZNF277 in the THP-1 cells infected with *Mtb* at different multiplicities of infection (MOI) via quantitative real-time PCR (qPCR). At the MOIs of 5 and 10, the intracellular levels of circ-ZNF277 increased by two-fold and three-fold, respectively, at 12 h post-infection (Figure 2). This experiment suggested that circ-ZNF277 responds to *Mtb* infection in THP-1 cells.

### 3.3. Overexpression of Circ-ZNF277 Inhibits Survival of Mtb in the THP-1 Cells

**Figure 3:**
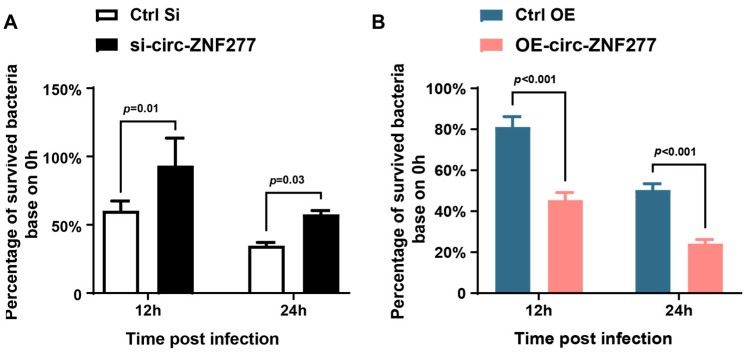
Overexpression of circ-ZNF277 inhibits *Mtb* survival in macrophages. (**A**) PMA-differentiated THP-1 cells transfected with siRNA against circ-ZNF277 (si-circ-ZNF277) or its negative controls, respectively, and then infected with *Mtb* at an MOI of 10 for 12 h. The *Mtb*-infected THP-1 cells were lysed with sterilized water at 0, 12, and 24 hpi for 25 min, 10-fold serially diluted with Hanks’ Balanced Salt Solution (HBSS), and plated on 7H11 plates. The viable intracellular survival of *Mtb* was detected by CFU counting; (**B**) The circ-ZNF277 overexpression lentivirus was used to generate stable circ-ZNF277-overexpressing THP-1 cell lines (OE-circ-ZNF277) or control THP-1 cells were differentiated with PMA and then infected with *Mtb* at an MOI of 10 for 12 h. The *Mtb*-infected THP-1 cells were lysed with sterilized water at 0, 12, and 24 hpi for 25 min, serially diluted 10-fold with HBSS, and plated on 7H11 plates. The viable intracellular survival of *Mtb* was detected by CFU counting. The data are expressed as mean ± SD. The statistically significant difference was analyzed with ANOVA for more than one comparison.

Further, we wanted to know the role of circ-ZNF277 during *Mtb* infection in THP-1 cells. To this end, the siRNA (si-circ-ZNF277) was transfected into THP-1 cells to knock down the expression of circ-ZNF277, and a lentivirus carrying circ-ZNF277 was used to generate a stable circ-ZNF277-overexpressing THP-1 cell line (OE-circ-ZNF277). Next, these THP-1 cells were infected with *Mtb* at an MOI of 10 for 12 h and 24 h. Afterwards, the live intracellular *Mtb* was determined using the colony-forming unit (CFU) assay. The CFU counting showed that the survival rate of *Mtb* in the circ-ZNF277-suppressed THP-1 cells was significantly increased compared with the controls at 12 and 24 h post-infection (*p* < 0.05) (Figure 3A). Conversely, the survival rate of *Mtb* in the circ-ZNF277-overexpressing THP-1 cells was significantly decreased compared with the controls (Ctrl-OE cells) (*p* < 0.001) (Figure 3B). Taken together, these experiments indicated that circ-ZNF277 inhibits the survival of *Mtb* within THP-1 cells.

### 3.4. The Expression Level of Circ-ZNF277 Affects NF-κB Signaling Pathway in the THP-1 Cells During Mtb Infection

**Figure 4:**
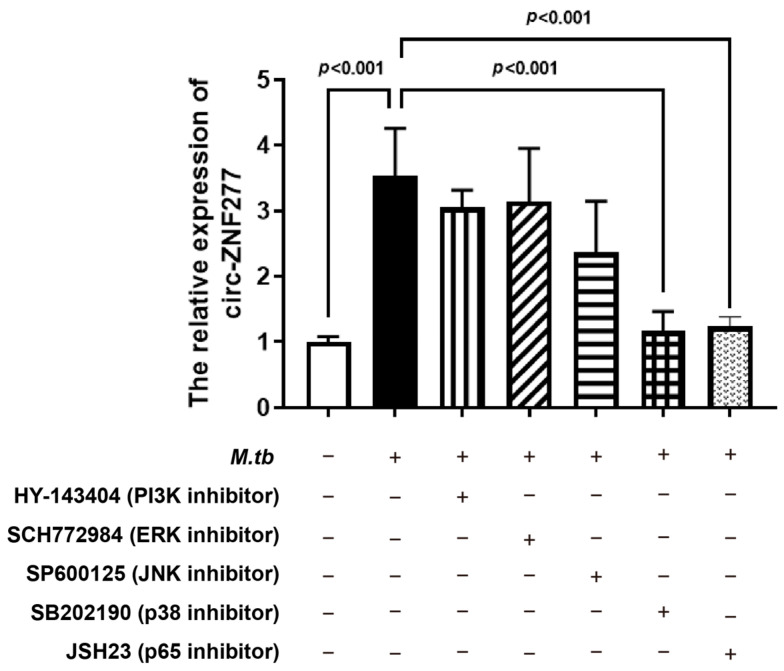
NF-κB and p38 MAPK signaling pathways involved in regulating the upregulation of circ-ZNF277 expression in *Mtb* infected THP-1 cells. PMA-differentiated THP-1 cells were treated with inhibitors of the PI3K (HY-143404), ERK (SCH772984), JNK (SP600125), p38 (SB202190), and NF-κB (JSH23) pathways for 2 h, and then infected with *Mtb* at an MOI of 10 for 12 h. At 12 h post-infection, circ-ZNF277 expression was detected by qPCR. The data are expressed as mean ± SD. The significant difference was analyzed using the Student’s *t* test. The different shapes were distinguished based on whether they were treated with these small molecule inhibitors or *Mycobacterium tuberculosis*, indicated by plus or minus signs.

NF-κB and MAPK signaling pathways were reported to be involved in the dysregulated expression of ncRNAs [49,53,54]. To find the possible signaling pathways which could affect the expression of circ-ZNF277 after *Mtb* infection, we treated the PMA-differentiated THP-1 macrophages with the signaling pathway inhibitors, including HY-143404, SCH772984, SP600125, SB202190, and JSH23, to/against PI3K, ERK, JNK, p38, and p65 pathways, respectively, for 2 h. Afterwards, the inhibitor-treated THP-1 macrophages were infected with *Mtb* at an MOI of 10 for 12 h, and then the relative expression levels of circ-ZNF277 was measured from each sample. The qPCR experiments showed that the expression levels of circ-ZNF277 from the THP-1 cells treated with HY-143404, SCH772984, or SP600125 inhibitors are similar to those from the untreated THP-1 cells during *Mtb* infection (Figure 4). Interestingly, circ-ZNF277 could not respond to *Mtb* infection in the THP-1 cells treated with SB202190 or JSH23. This suggests that NF-κB and p38 MAPK signaling pathways might affect the expression of circ-ZNF277 in the THP-1 cells during *Mtb* infection.

**Figure 5:**
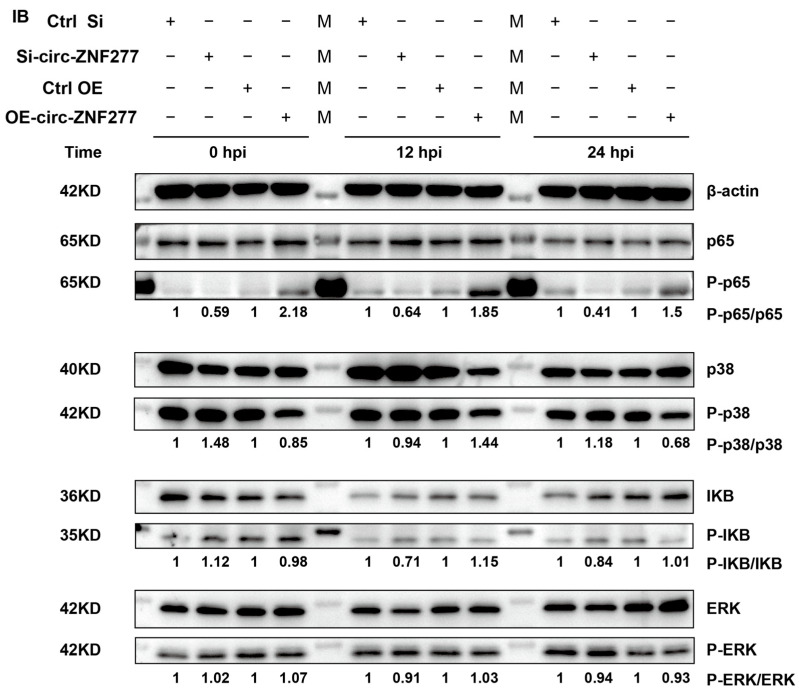
Overexpression of circ-ZNF277 promotes the phosphorylation of p65 in THP-1 cells. si-circ-ZNF277 transfected THP-1 cells and circ-ZNF277-overexpressing THP-1 cell lines, or their respective controls, were infected with *Mtb* at an MOI of 10 for 12 h. Total proteins were collected at designated time points. The expression levels of p65, p38, IκB, and ERK proteins and their respective phosphorylated proteins in *Mtb*-infected THP-1 cells were detected by a Western blot assay. The ratio of phosphorylated to unphosphorylated molecules was represented by the ratio of the intensity of bands and quantified by using Image J.

Further, we wanted to know whether the phosphorylation levels would be changed in NF-κB or p38 MAPK signaling pathways. Then, we used the phospho-specific antibodies to detect p65, p38, IκB, and ERK in the circ-ZNF277-suppressed and circ-ZNF277-overexpressed THP-1 cells infected with *Mtb* at an MOI of 10. After *Mtb* infection for 0 h, 12 h, and 24 h, the ratios of P-p65/p65 decreased in the circ-ZNF277-suppressed THP-1 cells, in contrast to those from the THP-1 cells transfected with the control sRNA. Conversely, the ratios of P-p65/p65 increased in the circ-ZNF277-overexpressed THP-1 cells infected with *Mtb* for 0 h, 12 h and 24 h. These suggested that the increased expression level of circ-ZNF277 promotes the phosphorylation level of p65 in the THP-1 cells during *Mtb* infection. However, the phosphorylation levels of p38, IKB, or ERK were not significantly changed in the circ-ZNF277-suppressed or circ-ZNF277-overexpressed THP-1 cells (Figure 5). Collectively, the expression level of circ-ZNF277 affected the phosphorylation level in the NF-κB signaling pathway during *Mtb* infection.

### 3.5. The Increased Expression Level of Circ-ZNF277 Promotes the Secretion of Inflammatory Factors in Mtb-Infected THP-1 Cells

**Figure 6:**
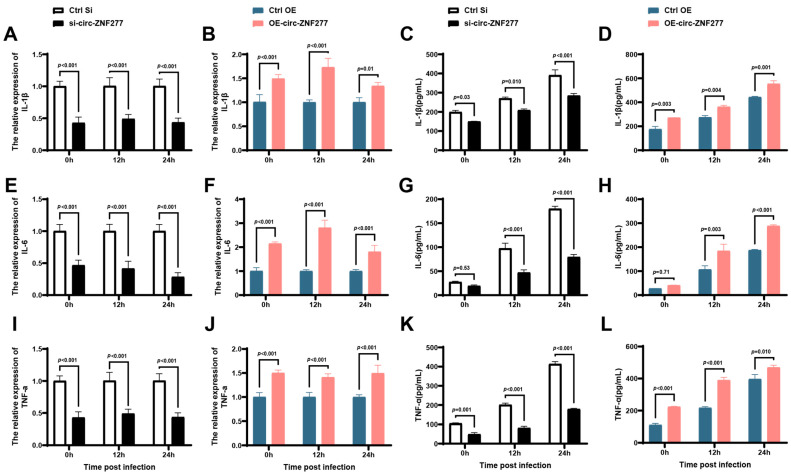
Circ-ZNF277 promotes secretion of inflammatory cytokines in the *Mtb*-infected macrophages. si-circ-ZNF277 transfected THP-1 cells and circ-ZNF277-overexpressing THP-1 cell lines, or their respective controls were infected with *Mtb* at an MOI of 10 for 12 h. Total RNA and supernatant were collected at designated time points. The transcription levels of IL-1β (**A**,**B**), IL-6 (**E**,**F**), and TNF-α (**I**,**J**) were detected by qPCR, and the protein levels of IL-1β (**C**,**D**), IL-6 (**G**,**H**), and TNF-α (**K**,**L**) were detected by ELISA. The data are expressed as mean ± SD. The statistically significant difference between the Ctrl si/si-circ-ZNF277 and Ctrl OE/OE-circ-ZNF277 groups at each time-point was analyzed with ANOVA.

The activation of NF-κB signaling pathway initiates the production of pro-inflammatory cytokines in macrophages, including IL-1, TNF-α, and IL-6 [55]. To examine whether circ-ZNF277 regulates the expression of pro-inflammatory cytokines after activating NF-κB pathway during *Mtb* infection, we detected the secretion of pro-inflammatory cytokines, including IL-1β, IL-6, and TNF-α in the THP-1 cells with suppressed or overexpressed circ-ZNF277 expression during *Mtb* infection by qPCR and ELISA. The qPCR experiment showed that the transcription levels of IL-1β, IL-6, and TNF-α in the circ-ZNF277 suppressed THP-1 cells were lower than those in the control THP-1 cells at 0, 12, and 24 h post-infection (*p* < 0.001) (Figure 6A,E,I). In contrast, the transcription levels of IL-1β, IL-6, and TNF-α were higher in circ-ZNF277 overexpressed THP-1 cells than those in the control THP-1 cells (*p* < 0.05) (Figure 6B,F,J). Similarly, the ELISA experiment showed that the secretion levels of IL-1β, IL-6 and TNF-α were decreased and increased in the circ-ZNF277 suppressed and circ-ZNF277 overexpressed THP-1 cells, respectively, during *Mtb* infection (Figure 6C,K). Taken together, these experiments indicate that the increased expression level of circ-ZNF277 promotes the secretion of IL-1β, IL-6, and TNF-α.

### 3.6. Circ-ZNF277 Specifically Targets miR-378d to Promote the Expression of Rab10

**Figure 7:**
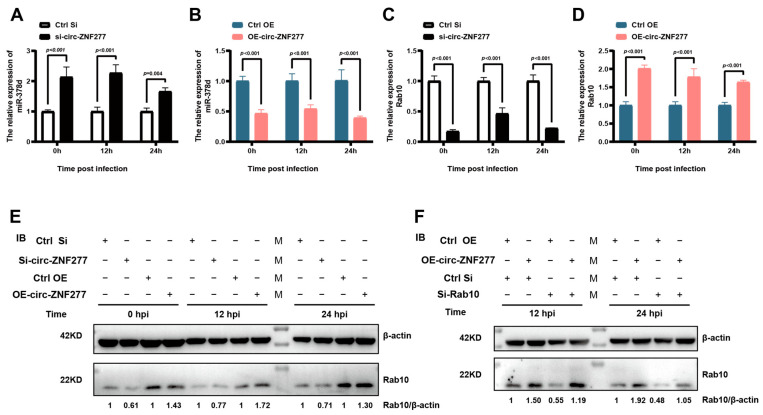
Circ-ZNF277 specifically targets miR-378d to promote Rab10 expression. si-circ-ZNF277 transfected-THP-1 cells and circ-ZNF277-overexpressing THP-1 cell lines, or their respective controls were infected with *Mtb* at an MOI of 10 for 12 h. Total RNA and proteins were collected at designated time points. The transcription level of miR-378d (**A**,**B**) and Rab10 (**C**,**D**) were detected by qPCR; (**E**) The protein levels of Rab10 were detected by Western blot; (**F**) Circ-ZNF277-overexpressing THP-1 cell lines or control THP-1 cells were differentiated with PMA and then transfected with si-Rab10 or its control, followed by infection with *Mtb* at an MOI of 10 for 12 h. The protein levels of Rab10 were detected by Western blot. The data are expressed as mean ± SD. The significant difference was analyzed using the Student’s *t* test for one comparison or ANOVA for more than one comparison.

Since miR-378d inhibits the expression of Rab10 [49], we wanted to know if circ-ZNF277 could target miR-378d to affect the expression of Rab10. RT-PCR experiments showed that inhibiting the expression of circ-ZNF277 in THP-1 cells during *Mtb* infection leads to increased transcription levels of miR-378d (Figure 7A) and decreased transcription levels of Rab10 (Figure 7C). Conversely, overexpressing circ-ZNF277 in THP-1 cells during *Mtb* infection leads to decreased transcription levels of miR-378d (Figure 7B) and increased transcription levels of Rab10 (Figure 7D), consistent with the changes on expression levels of Rab10 observed by Western blot experiments (Figure 7E). However, Rab10 siRNA (si-Rab10) could offset the effort of overexpressing circ-ZNF277 on the expression levels of Rab10 in the THP-1 cell during *Mtb* infection (Figure 7F). Collectively, these findings suggest that circ-ZNF277 acts as a sponge of miR-378d to counteract its suppression on the expression of Rab10.

### 3.7. Circ-ZNF277 Promotes the Secretion of Inflammatory Cytokines to Inhibit the Survival of Mtb in THP-1 Cells via Circ-ZNF277/miR-378d/Rab10 Axis

**Figure 8:**
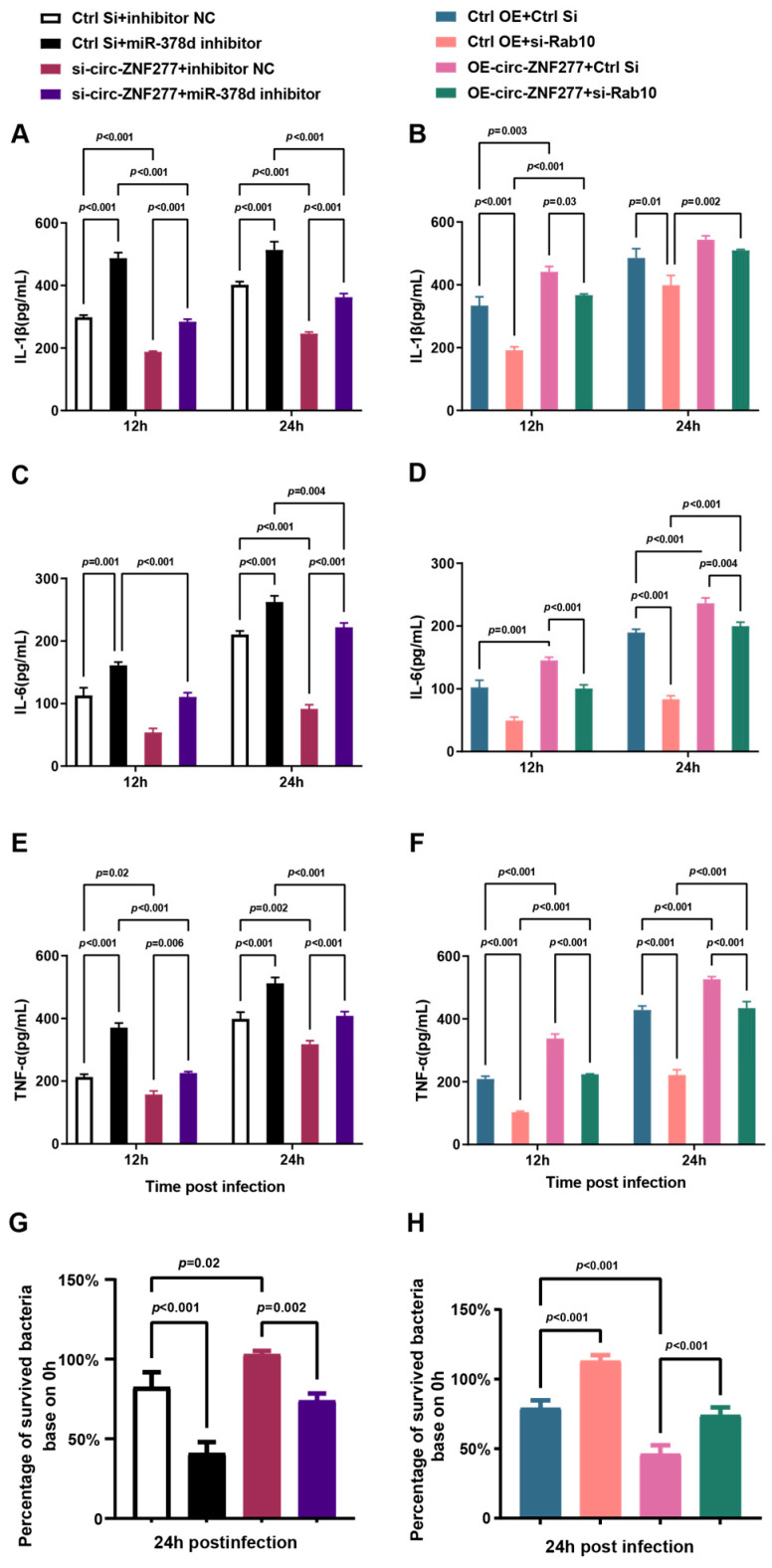
Circ-ZNF277 promotes secretion of inflammatory cytokine and inhibits *Mtb* intracellular survival via miR-378d/Rab10. (**A**,**C**,**E**,**G**) PMA-differentiated THP-1 cells were co-transfected with si-circ-ZNF277 and miR-378d inhibitor or their respective controls, and then infected with *Mtb* at an MOI of 10 for 12 h. The supernatant of *Mtb*-infected THP-1 cells were filtered using a 0.22 μm filter at 12 and 24 hpi, and the secretion of IL-1β, IL-6, and TNF-α was detected by ELISA. The intracellular survival of *Mtb* was detected by CFU counting. *Mtb* infected THP-1 cells were lysed by sterilized water at 0 and 24 hpi for 25 min, 10-fold serially diluted with HBSS, and plated on 7H11 plates. (**B**,**D**,**F**,**H**) Circ-ZNF277-overexpressing THP-1 cell lines or control THP-1 cells were differentiated with PMA and then transfected with si-Rab10 or its control, followed by infection with *Mtb* at an MOI of 10 for 12 h. The supernatant of *Mtb* infected THP-1 cells was filtered using a 0.22 μm filter at 12 and 24 hpi, and the secretion of IL-1β, IL-6, and TNF-α was detected by ELISA. For CFU counting, *Mtb* infected THP-1 cells were lysed by sterilized water at 0 and 24 hpi for 25 min, 10-fold serially diluted with HBSS, and plated on 7H11 plates. The data are expressed as mean ± SD. The significant difference was analyzed using the Student’s *t* test for one comparison or ANOVA for more than one comparison.

Next, we wanted to know whether THP-1 macrophages secrete pro-inflammatory cytokines for inhibiting the survival of *Mtb* in a manner regulated by the circ-ZNF277/miR-378d/Rab10 axis. PMA-differentiated THP-1 cells were co-transfected with si-circ-ZNF277 and the inhibitor of miR-378d, a single-stranded RNA molecule, and then infected with *Mtb* at an MOI of 10 for 12 h. The secretion of IL-1β, IL-6, and TNF-α was detected using ELISA, while the intracellular survival of *Mtb* was assessed by CFU counting 24 h post-infection. Inhibiting circ-ZNF277 expression indeed reduced the extracellular levels of IL-1β, IL-6, or TNF-α and increased the intracellular survival of *Mtb* in THP-1 cells (Figure 8A,C,E,G). Meanwhile, in the presence of the miR-378d inhibitor, the secretion levels of IL-1β, IL-6, or TNF-α, as well as the intracellular survival of *Mtb* in the circ-ZNF277-suppressed THP-1 cells, were not significantly different compared to that observed in the THP-1 cells infected with the unrelated siRNA plus the unrelated inhibitor control. This indicated that the miR-378d inhibitor could significantly reduce the effect of circ-ZNF277 knockdown on the secretion of inflammatory cytokines and the survival of *Mtb* in vivo (Figure 8A,C,E,G). Additionally, the PMA-differentiated circ-ZNF277-overexpressing THP-1 cell lines were used for the transfection with si-Rab10, a siRNA against Rab10. Overexpression of circ-ZNF277 increased the secretion of IL-1β, IL-6, and TNF-α and decreased the survival of *Mtb* in THP-1 cells infected with *Mtb* at an MOI of 10 for 12 h (Figure 8B,D,F,H). Conversely, in the presence of si-Rab10, the secretion of IL-1β, IL-6, and TNF-α, as well as the survival of *Mtb* in the circ-ZNF277-overexpressing THP-1 cells, were not significantly different compared to those observed in the THP-1 cells infected with the control siRNA (Figure 8B,D,F,H). This indicated that si-Rab10 significantly offsets the effect of overexpression of circ-ZNF277 on the secretion of inflammatory cytokines and the survival of *Mtb* in vivo. Taken together, these experiments suggested that circ-ZNF277 promotes the secretion of inflammatory cytokines IL-1β, IL-6, and TNF-α to inhibit the survival of *Mtb* in THP-1 cells via circ-ZNF277/miR-378d/Rab10 axis.

## 4. Discussion

In the *Mtb*-infected host cells, circRNAs showed extensive differential expression [28,51,56]. However, only a few circRNAs present in the cytoplasm were verified to regulate the expression of the genes involved in macrophage autophagy and polarization-related biological processes during pathogens infection. In this study, we identified a novel circRNA molecule, namely circ-ZNF277, which shows upregulated expression in the *Mtb*-infected macrophages. Circ-ZNF277 is mainly localized in the cytoplasm of THP-1 cells. It acts as a competing endogenous RNA (ceRNA) to relieve miR-378d-mediated inhibition of Rab10, thereby promoting the secretion of pro-inflammatory cytokines (IL-1β, IL-6, and TNF-α) and contributing to the clearance of intracellular *Mtb*. Similarly, in the detection of circular RNA expression, circAGFG1 expression was found to be upregulated in macrophages of patients with active tuberculosis. Different from the circ-ZNF277/miR-378d/Rab10 pathway, CircAGFG1 inhibits apoptosis and enhances macrophage autophagy via miRNA-1257/Notch2 axis [57]. In addition, some other circular RNAs, such as circ-0045474, circTRAPPC6B, and circ-0001490, exhibited significantly downregulated expression in the host cells during *Mtb* infection [39,40,58,59,60,61]. Circ-0045474 decreased in monocytes of TB patients and it may inhibit *Mtb*-induced macrophage autophagy by promoting TNKS2 expression via miR-582-5p inhibition [39]. However, it is not clear whether circ-0045474 affects *Mtb* intracellular survival. Also, circTRAPPC6B exhibits downregulated expression in the peripheral blood mononuclear cells (PBMCs) of patients with active tuberculosis. CircTRAPPC6B could increase ATG16L1 expression by competitively binding miR-874-3p, enhancing the formation of the ATG16L1-ATG12-ATG5 complex and ultimately promoting autophagosome formation and autophagy to clear intracellular *Mtb* [40]. In addition, circTRAPPC6B could induce the expression of IL-6 and IL-1β by competitively binding miR-874-3p, thus clearing intracellular *Mtb* [62]. This indicates that the downregulation of circTRAPPC6B induced by *Mtb* infection might be one of the strategies for *Mtb* to evade host cell autophagy and pro-inflammatory cytokine killing, thereby surviving in macrophages. Collectively, these findings suggest that circRNAs are indeed multifaceted in regulating immune responses during *Mtb* infection.

The functional differences of circ-ZNF277, circTRAPPC6B, and circ-0045474 in *Mtb* infection might be related to the dysregulated expression of different functional target genes through the ceRNA mechanism. In this study, circ-ZNF277 enhances Rab10 expression by specifically binding miR-378d. The Rab GTPase family proteins are the key components in specific biological processes of immune cells such as macrophages. These proteins control processes like cell migration, phagocytosis, endocytosis, and exocytosis through various adaptor proteins, including coat proteins, phospholipids, kinases, phosphatases, molecular motors, actin, or microtubule cytoskeletons [63]. Rab10 is primarily localized in early endosomes and the Golgi apparatus and it is involved in Golgi-to-endosome transport and promotes the replication of Legionella pneumophila [64]. Additionally, Rab10 is highly expressed in macrophages in an in vivo model of LPS-induced acute lung injury, regulating TLR4 transport from the Golgi to the plasma membrane and affecting the severity of lung injury [65]. During *Mtb* infection, Rab10 regulates the transition of nascent phagosomes to early endosomes and the maturation of *Mtb*-containing phagosomes [66]. Here, we found that the higher expression level of Rab10 in macrophages could promote the secretion of pro-inflammatory cytokines (IL-6, IL-1β, and TNF-α), thereby helping to clear intracellular *Mtb*. However, whether the higher expression level of Rab10 could promote *Mtb* recognition and the maturation of *Mtb*-containing phagosomes for the clearance of the intracellular *Mtb* needs to be further investigated. Additionally, Rab10 could be regulated by other circRNAs, such as circ-TDRD9 [67,68]. Circ-TDRD9 enhances the expression of Rab10 through the miR-223-3p/Rab10 axis, promoting acute lung injury induced by sepsis [68]. However, we did not observe the abnormal expression for circ-TDRD9 in the hosts during *Mtb* infection. These suggest that (i) circ-TDRD9 and circ-ZNF277 have different roles in regulating the expression of Rab10 and (ii) circ-ZNF277 is specifically associated with *Mtb* infection.

The NF-κB signaling pathway is crucial in the host’s immune response to *Mtb* invasion [69]. Here, we found that the p65-NF-κB signaling pathway regulates the upregulated expression of circ-ZNF277 induced by *Mtb* infection. *Mtb* activates NF-κB signaling pathway not only through the classic TLR2/MyD88/NF-κB pathway but also by inducing the dysregulated expression of various ncRNAs [70]. For example, microRNA Let-7f promotes the activation of NF-κB signaling pathway, while miR-18b inhibits the activation of NF-κB signaling pathway during *Mtb* infection [50,71]. Similarly, circ-ZNF277 inhibits the phosphorylation of the p65 molecule, indicating a reciprocal regulatory relationship between the NF-κB signaling pathway and circ-ZNF277. However, the exact mechanism needs to be further investigated.

**Figure 9:**
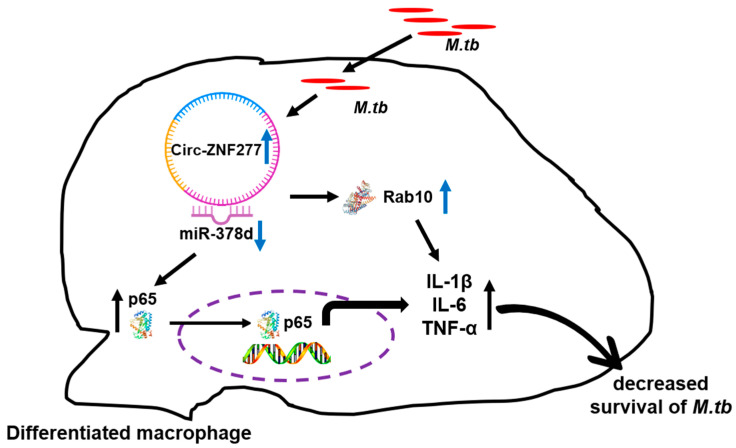
Model for the roles of circ-ZNF277/miR-378d/Rab10 axis in *Mtb*-macrophage interaction. The Circular RNA ZNF277 is upregulated during *Mtb* infection. Circ-ZNF277 could bind microRNA-378d (miR-378d), blocking miR-378d’s inhibitory effect on Rab10, activating the NF-κB signaling pathway, and increasing the secretion of pro-inflammatory cytokines (IL-1β, IL-6, and TNF-α). This ultimately inhibits the survival of *Mtb* in THP-1 cells.

Overall, we demonstrated that circ-ZNF277 specifically sponges miR-378d to unblock the inhibitory effect of miR-378d to Rab10, therefore activating NF-κB signaling pathway and enhancing the secretion of pro-inflammatory cytokines including IL-1β, IL-6, and TNF-α. This ultimately leads to the reduced survival of *Mtb* in THP-1 cells (Figure 9). Our study provides novel insights into circular RNAs regulating immune responses in the host during pathogens infection.

## Data Availability

The datasets generated in this study are available upon request from the corresponding authors.

## Supplementary Materials

The following supporting information can be downloaded at: https://www.mdpi.com/article/10.3390/cells14040262/s1, Table S1. siRNA and RNA oligonucleotides sequences. Table S2. Primers for RT-PCR and qPCR. Table S3. Antibody used in this study. Supplementary Figure S1. Potential existence of circ-ZNF277 in THP-1 cells.
