# Supplementary material for: Circular RNA ZNF277 Sponges miR-378d to Inhibit the Intracellular Survival of Mycobacterium tuberculosis by Upregulating Rab10
Source: Cells. 2025 Feb 12;14(4):262. doi: 10.3390/cells14040262 (PMC11853707; doi:10.3390/cells14040262)
Supplement: Supplementary file 1 [file cells-14-00262-s001.zip › cells-3447094-supplementary.pdf]

Table S1. siRNA and RNA oligonucleotides sequences

Sequence Name	Sequences (5′- 3′)
hsa-si-circ-ZNF277	sense: CAAAGACAGUAAGGAUUGUTT<br>antisense: ACAAUCCUUACUGUCUUUGTT
hsa-si-Rab10	sense: GGGUAUCAUGCUAGUAUAUTT<br>antisense: AUAUACUAGCAUGAUACCCTT
hsa-miR-378d mimic	sense:<br>UGAGGGGCCUCAGACCGAGCUUUU<br>antisense: UCUGACUCCAAGUCCAGUUU
hsa-miR-378d inhibitor	UUUCUGACUCCAAGUCCAGU

Table S2. Primers for RT-PCR and qPCR

Primer Name	Sequence (5′- 3′)	Product s (bp)
hsa-β-actin	F: CATGTACGTTGCTATCCAGGC<br>R: CTCCTTAATGTCACGCACGAT	250
hsa-circ-DCLRE1C<br>-<br>Divergent	F: TTGGAGAAAGGAGCAGAAAA<br>R: ATCACAGAACGTAGTATCCA	185
hsa-circ-CRIM1-<br>Divergent	F: CCCGCATAGTCTCTCGTGG<br>R: CAAAGGGATTGCTGCAGGTT	192
hsa-circ-ZNF277-<br>Divergent	F: GCTGATTTCCAAAGACAGTAAGGA<br>R: TGTCTTGTTCAGCCACAGGAA	143
hsa-circ-ZNF277-<br>Convergent	F: GAGTTGCTGGACCATCAGGAAG<br>R: CCTCCATGTGGACATACAACTTC	123
Universal Reverse	GCTGTCAACGATACGCTACGTAAC	
U6	F: CTCGCTTCGGCAGCACA<br>R: AACGCTTCACGAATTTGCGT	
hsa-miR-378d	ACTGGACTTGGAGTCAGAAA	
hsa-IL-1β	F: GTGGCAATGAGGATGACTTGTTC<br>R: GGTGGTCGGAGATTCGTAGCT	120
hsa-IL-6	F: ACTCACCTCTTCAGAACGAA<br>R: CCATCTTTGGAAGGTTCAGG	149
hsa-TNF-α	F: GGAGAAGGGTGACCGACTCA<br>R: CTGCCCAGACTCGGCAA	70
hsa-Rab10	F: TGGAACTACAAGGAAAGAAGAT<br>R: TAGTAGGAGGTTGTGATGGT	78

Table S3. Antibody used in this study

Antibody	Company
β-actin (Cat no. 60008-1)	Protech, Wuhan, China
rabbit mAb to NF-κB p65(E379) (ab16502)	Abcam, MA, USA
rabbit mAb to phosphor-NF-κB p65 (ab109458)	Abcam, MA, USA
rabbit mAb to p44/42 MAPK (Erk1/2) (Cat no. 4695)	Cell signaling, MA, USA
rabbit mAb to phospho-p44/42 MAPK (Erk1/2) (Cat no. 4370)	Cell signaling, MA, USA
rabbit mAb to p38 alpha/MAPK14 (ab182453)	Abcam, MA, USA
rabbit mAb to phosphor-p38 alpha/MAPK14 (ab178867)	Abcam, MA, USA
rabbit mAb to IKB alpha (ab32518)	Abcam, MA, USA
rabbit mAb to phosphor-IKB alpha (ab133462)	Abcam, MA, USA
rabbit mAb to Rab10 (ab181367)	Abcam, MA, USA

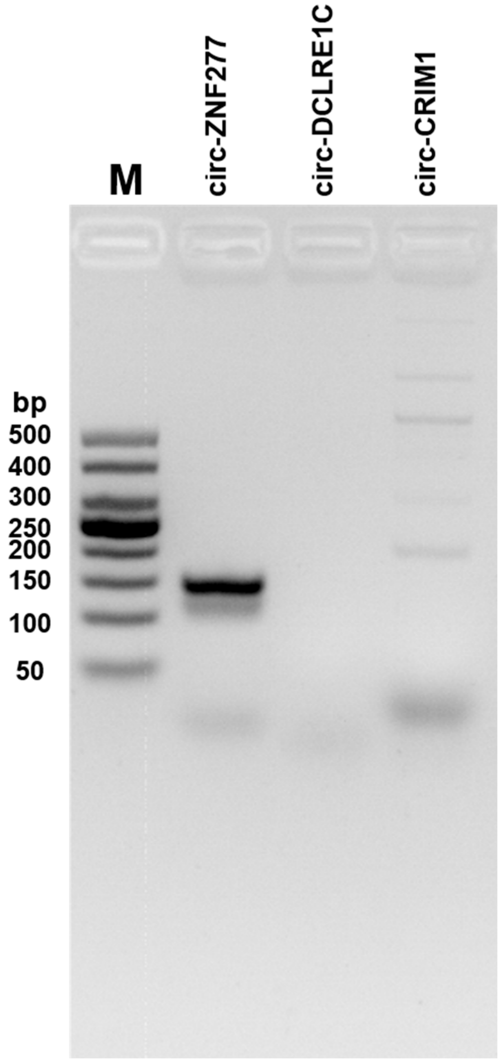

Supplementary Figure S1. Potential existence of circ-ZNF277 in THP-1 cells. circ-ZNF277, circ-DCLRE1C, and circ-CRIM1 were amplified from the cDNA of THP-1 cells using divergent primers in RT-PCR experiments. The amplification products for circ-ZNF277, circ-DCLRE1C, and circ-CRIM1 were 143 bp, 185 bp, and 192 bp in length, respectively.
